# A hierarchical estimator development for estimation of tire-road friction coefficient

**DOI:** 10.1371/journal.pone.0171085

**Published:** 2017-02-08

**Authors:** Xudong Zhang, Dietmar Göhlich

**Affiliations:** Product Development Methods and Mechatronics, Technical University of Berlin, Berlin, Germany; Beihang University, CHINA

## Abstract

The effect of vehicle active safety systems is subject to the friction force arising from the contact of tires and the road surface. Therefore, an adequate knowledge of the tire-road friction coefficient is of great importance to achieve a good performance of these control systems. This paper presents a tire-road friction coefficient estimation method for an advanced vehicle configuration, four-motorized-wheel electric vehicles, in which the longitudinal tire force is easily obtained. A hierarchical structure is adopted for the proposed estimation design. An upper estimator is developed based on unscented Kalman filter to estimate vehicle state information, while a hybrid estimation method is applied as the lower estimator to identify the tire-road friction coefficient using general regression neural network (GRNN) and Bayes' theorem. GRNN aims at detecting road friction coefficient under small excitations, which are the most common situations in daily driving. GRNN is able to accurately create a mapping from input parameters to the friction coefficient, avoiding storing an entire complex tire model. As for large excitations, the estimation algorithm is based on Bayes' theorem and a simplified “magic formula” tire model. The integrated estimation method is established by the combination of the above-mentioned estimators. Finally, the simulations based on a high-fidelity CarSim vehicle model are carried out on different road surfaces and driving maneuvers to verify the effectiveness of the proposed estimation method.

## Introduction

Many advanced vehicle control systems, such as the anti-lock braking system (ABS), the acceleration slip regulation (ASR), and the electronic stability programming (ESP), have become standard equipment on automobiles nowadays to guarantee the vehicle stability under critical conditions [1–3]. The performance of them relies heavily on the accurate knowledge of tire-road friction coefficient (TRFC). For example, electronic stability programming (ESP) can precisely compute the control boundary with the awareness of TRFC in order to make full use of available traction and braking torque. Besides, regarding adaptive cruise control system a known friction coefficient enables it to make the braking decision timely and accurately. However for the reason of technical or cost, such an important parameter cannot be directly measured. Therefore, in order to obtain a relatively ideal dynamic control effect, the TRFC should be estimated precisely and robustly.

Quite a few studies have been carried out to work out different estimation approaches. Generally speaking, these estimation methods are mainly classified into two categories: “cause-based” and “effect-based” approaches [4]. “Cause-based” approaches estimate the TRFC by detecting road coverings (water, snow, ice, oil etc.) using special sensors like optical camera and temperature sensors, etc. F. Holzmann [5] proposed a predictive methodology for the estimation of friction coefficient by using a camera and a microphone. The road surface is deduced through matching the current specimen with the prestored specimens. In [6], a method for detection of ice formation on road surfaces was presented. It used infrared thermometers to detect heat energy emitted during freezing, which was verified in field conditions. These caused-based methods make it possible to estimation TRFC without physical excitation. However, these friction condition recognition methods are conducted only from the aspect of road conditions. The other factors such as tire state (new or worn, winter tire or summer tire) or tire pressure are not taken into account.

“Effect-based” approaches use vehicle and tire dynamic effects such as tire-tread deformation, vehicle dynamics, and so on. Tire-tread deformation measurement relies heavily on the sensor capability. Therefore it is difficult to be applied on production vehicles due to the cost and the technical challenges of the sensors [7]. Resulting from the fairly easy and cost-effective implementation, the estimation approaches using vehicle dynamic response information has drawn increasing interest in recent years [8–12]. In [13], an estimation method of TRFC was introduced based on extend Kalman filter and neural network. Simulation results show that under uncritical driving conditions it has a good performance. Y. J. Hsu and J. Gerdes [14] proposed an algorithm to obtain the friction coefficient using readily available steering torque information and measured sideslip angle from GPS device. G. Xsin [15] presented a maximum TRFC estimator by comparing the samples of the estimated TRFC with the standard TRFC of each typical road, and using the minimum statistical error as the recognition principle to improve identification robustness. Aiming at four-wheel independently actuated electric vehicles, a TRFC estimation method was developed with the assistance of the additional yaw moment induced by the longitudinal tire force difference [16, 17].

In this study, the presented hierarchical estimation method focuses on the dynamic characteristics of a four-motorized-wheels electric vehicle to achieve the TRFC estimation and contributes in the following aspects: first, an estimator based on unscented Kalman filter (UKF) is applied to identify vehicle motion states as well as tire forces. These estimated values are used as inputs of the TRFC estimation algorithm. Subsequently, according to the different levels of dynamic excitation, a hybrid TRFC estimator is developed by means of artificial neural network (ANN) and Bayesian theorem. Finally, the vehicle state estimator and TRFC estimator are able to simultaneously communicate and correct each other throughout the whole estimation process.

This paper proceeds as follows. Section 2 presents a mathematical vehicle dynamic model. Estimation algorithms including vehicle state estimation and TRFC estimation are described in section 3. The results of computer simulation are shown and analyzed in Section 4. At last, Section 5 concludes this paper.

## Vehicle modeling

The section presents a 3-DOFs vehicle motion model, which serves as a basis of the UKF estimator. Subsequently, in order to estimate longitudinal and lateral tire forces, the wheel dynamics equation and “Pacejka 2002” tire model are used.

### Vehicle motion submodel

There exists a contradictory relationship between the complexity of vehicle dynamics model and performance of the estimator. A model with high DOF is precise and contains more dynamic information, however requires a larger number of parameters that are difficult to acquire. If inappropriate parameters are used, the model containing high DOF would generate further errors when compared to the model with low DOF. Therefore, there is a need of balance and compromise between the modelling complexity and estimator performance. This paper has selected the 3 DOFs vehicle model as the basis of the estimator mainly by considering the following aspects:

1. The vertical DOFs of the vehicle body and suspension system mainly affect the vehicle smoothness and comfort but have little effect on the vehicle stability. Thus they are consciously omitted in the mathematical modelling.
2. The normal load of each tire is constantly altered, which is caused by the lateral and longitudinal vehicle load transfer during the steering, acceleration and deceleration. Meanwhile, the tire normal load has a directly effect on tire cornering stiffness and longitudinal stiffness, which also leads to the control boundary changing of the controller [10, 18]. Therefore, it is very essential to take into account the load transfer for a better analysis about the vehicle characteristics. Two main reasons may lead to vehicle load transfer: one is the inertial force generated by the longitudinal or lateral acceleration. It is the decisive factor that causes whole vehicle load transfer; the other is the pitch and the roll dynamics that results in the change of center of gravity and causes load transfer. It has very little influence on the vehicle load transfer. As a consequence, during the development of the estimator, we should ignore the pitch and roll dynamics and only focus on the load transfer due to the inertial force in order to improve the computational efficiency.

From the reasons discussed above, the vehicle modelling starts from a vehicle motion submodel with 3 DOF, the longitudinal velocity *u*, the lateral velocity *v*, and the yaw rate *r*. It is assumed that the vehicle is moving on a flat horizontal plane. Additionally, the vertical, roll and pitch dynamics are omitted in order to reduce the state variables and computational effort.

**Fig 1:**
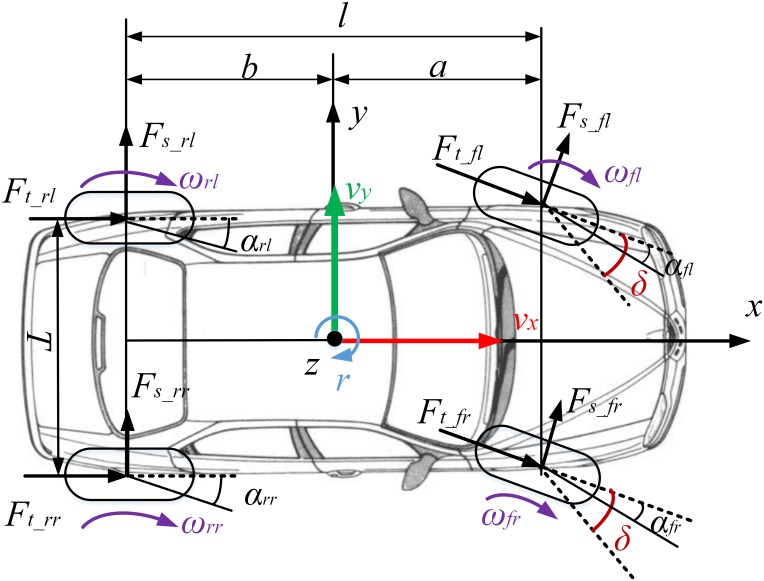
Schematic diagram of a vehicle model.

Fig 1 shows a typical schematic diagram of vehicle model. The wheel positions is numbered with the subscript *ij = fl*,*rl*,*fr*,*rr* denoting front left, rear left, front right and rear right respectively.

The vehicle motion equations can be expressed as follows. Longitudinal and lateral motions along the *x* and *y*-axis:

$$m\cdot a_{x}=\sum F_{x\_ij}+\frac{1}{2}C_{D}A_{f}\rho u^{2}$$ (1)

$$m\cdot a_{y}=\sum F_{y\_ij}$$ (2)

Rotational motions of yaw about z-axis:

$$I_{z}\cdot \dot{r}=a\left(F_{y\_fl}+F_{y\_fr}\right)-b\left(F_{y\_rl}+F_{y\_rr}\right)+T/2\left(F_{x\_fl}+F_{x\_rl}\right)-T/2\left(F_{x\_fr}+F_{x\_rr}\right)$$ (3)

where *a*_*x*_ is the longitudinal acceleration; *a*_*y*_ is the lateral acceleration and *r* is the yaw rate; *C*_*d*_, *A* and *ρ* denote the air resistance coefficient, the frontal projected area and the air density, respectively. Moreover, the acceleration terms are defined as

$$a_{x}=\dot{u}-vr$$ (4)

$$a_{y}=\dot{v}+ur$$ (5)

The forces *F*_*x_ij*_ and *F*_*y_ij*_ are the tire forces along the x and y axis, which could be expressed as functions of the tire longitudinal and lateral forces by the following equations

$$F_{x\_ij}=F_{t\_ij}\cdot cos\;\delta_{T\_ij}-F_{s\_ij}\cdot sin\;\delta_{T\_ij}$$ (6)

$$F_{y\_ij}=F_{t\_ij}\cdot sin\;\delta_{T\_ij}+F_{s\_ij}\cdot cos\;\delta_{T\_ij}$$ (7)

where *δ*_*T_ij*_ is the steering angle of wheel *ij*; *F*_*t_ij*_ and *F*_*s_ij*_ denote the longitudinal and lateral forces of tire.

According to the longitudinal and lateral load transfer, the normal load expressions can be written as

$$F_{z\_fl}=mg\frac{b}{2l}-ma_{x}\frac{h}{2l}+ma_{y}\frac{b}{l}\frac{h}{T}$$ (8)

$$F_{z\_rl}=mg\frac{b}{2l}+ma_{x}\frac{h}{2l}+ma_{y}\frac{a}{l}\frac{h}{T}$$ (9)

$$F_{z\_fr}=mg\frac{b}{2l}-ma_{x}\frac{h}{2l}-ma_{y}\frac{b}{l}\frac{h}{T}$$ (10)

$$F_{z\_rr}=mg\frac{b}{2l}+ma_{x}\frac{h}{2l}-ma_{y}\frac{a}{l}\frac{h}{T}$$ (11)

### Wheel dynamics

As for the 4-motorized-wheels EV, the torque signal of each tire can be measured directly. Thus the longitudinal force can be calculated by the rotational dynamic equation instead of complicated tire models, which is shown below:

$$F_{t\_ij}=\frac{T_{m\_ij}\cdot beta-J_{w}\cdot {\dot{w}}_{ij}}{R},\;ij=fl,\;fr,\;rl,\;rr$$ (12)

where *w*_*ij*_ is the wheel rotational speed; *J*_*w*_ is the wheel rotational inertia; *beta* is the transmission ratio; *T*_*m_ij*_ denotes the motor torque output. Besides, *R* is the tire loaded radius and in this study it is assumed to be a constant.

### “Pacejka 2002” tire model

In this study, the well-known semi-empirical “Pacejka 2002” tire model [19] is employed for lateral tire force calculation and also for the artificial neural network data collecting. The difference of the two applications is that for lateral tire force calculation, only the related parameters and equations are used, which reduces the computational effort, however all the parameters and equations involved in tire model are taken into consideration for artificial neural network training.

Longitudinal and lateral forces are calculated by “Pacejka 2002” in two steps. First for pure slip condition [19]:

$$F_{t0}=D_{x}sin\left\{C_{x}\;arctan\left\{B_{x}\lambda -E_{x}\left[B_{x}\lambda -arctan\left(B_{x}\lambda \right)\right]\right\}\right\}+S_{Vx}$$ (13)

$$F_{s0}=D_{y}\;sin\left\{C_{y}\;arctan\left\{B_{y}\alpha -E_{y}\left[B_{y}\alpha -arctan\left(B_{y}\alpha \right)\right]\right\}\right\}+S_{Vy}$$ (14)

Subsequently for the combined slip condition [19]:

$$F_{t}=G_{x}F_{t0}$$ (15)

$$F_{s}=G_{y}F_{s0}+S_{Vyk}$$ (16)

where *G*_*x*_ and *G*_*y*_ are the weighting functions if the longitudinal and lateral force for pure slip which always have the values between 0 and 1. The lateral and longitudinal slip ratio of each tire are given as

$$\alpha_{ij}=\delta_{f}-arctan\left(\frac{\;v+ar}{\;u\pm \frac{1}{2}Tr}\right),\;ij=fl,\;fr$$ (17)

$$\alpha_{ij}=-arctan\left(\frac{\;v-br}{\;u\pm \frac{1}{2}Tr}\right),\;ij=rl,\;rr$$ (18)

$$\lambda_{ij}=-\frac{u_{w_{ij}}-R\cdot \omega_{ij}}{u_{w_{ij}}},\;ij=fl,\;fr,\;rl,\;rr$$ (19)

The wheel center speed *u*_*w_ij*_ is given by

$$u_{w\_ij}=\left(u\pm \frac{1}{2}Tr\right)cos\delta_{f}+\left(\;v+ar\right)sin\delta_{f},\;ij=fl,\;fr$$ (20)

$$u_{w\_ij}=u\pm \frac{1}{2}Tr,\;ij=rl,\;rr$$ (21)

For the sake of simplicity, the wheel camber is neglected as a low-effect parameter. Additionally, the self-aligning torque also is not taken into account, for the artificial network aims at establish the relationship only between longitudinal and lateral tire forces and road friction coefficient.

## Hierarchical estimation algorithm design

**Fig 2:**
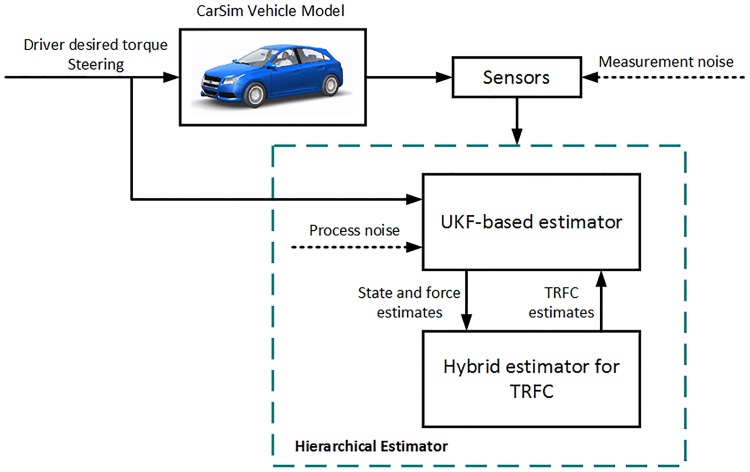
Block diagram of the proposed hierarchical estimator.

The block diagram of Fig 2 shows the hierarchical estimation system. The driver desired torque and steering angle are the control input for the detailed vehicle model in CarSim and the UKF estimator. Moreover, the CarSim model also provides the measurement input of the wheel rotational speed, the yaw rate, the longitudinal and lateral acceleration. Since motor torque and wheel rotational speed signals can be directly acquired from the motor controller, the longitudinal tire force is calculated based on Eq (12). According to Eq (14) of “Pacejka 2002” tire model with an initial friction coefficient 0.8, after identifying the vehicle motion states ***x*** *= [u*, *v*, *r]*^*T*^ using UKF estimator, the lateral tire force is also obtained. These estimated values from UKF estimator are used as the inputs of the hybrid TRFC estimator. Meanwhile, the friction coefficient, which is the output of the hybrid TRFC estimator, in turn is taken as the input of the UKF estimator. Throughout the whole estimation process, the two estimators simultaneously communicate and correct each other to accurately achieve combined state and TRFC estimation.

### Vehicle state estimation based on UKF

According to the vehicle model described in section 2, the dynamic system can be rewritten in the form of discretization nonlinear transition equation as follows

$$\begin{array}{l} x\left(k\right)=f\left(x\left(k-1\right),\;u\left(k-1\right),w\left(k-1\right)\right)\\ y\left(k\right)=h\left(x\left(k-1\right),v\left(k-1\right)\right) \end{array}$$ (22)

where ***x****(k)* is the state at the sampling time *k*, ***x*** *= [u*, *v*, *r]*^*T*^; ***u****(k)* is the input to the system at the sampling time *k*, ***u*** *= [δ*, *T*_*m_ij*_*]*^*T*^; ***y****(k)* is a set of measurement output, ***u*** *= [a*_*x*_, *a*_*y*_, *r*, *w*_*ij*_*]*^*T*^. Besides, ***w*** and ***v*** are the process noise and measurement noise vectors, assuming to be white Gaussian uncorrelated noises.

For the state estimation of nonlinear system, the extended Kalman filter (EKF) is a widely used approach [20]. However the main drawback of the EKF is Jacobian matrices calculation, which requires costly computation. Besides, EKF only employs the first order Taylor expansion on nonlinear system, which may lead to great error or even divergence of the filter if the model is serious nonlinear. Addressing these issues, the UKF utilizes a deterministic sampling technique known as the unscented transform (UT) to pick a minimal set of sample points (called sigma points) around the mean, which is a derivative-free alternative to EKF and meanwhile avoids the expensive update of the Jacobian matrix on each iteration. Additionally, UKF achieves third order Taylor series expansion accuracy [21]. Thus in this study, UKF is applied and elaborated as follows.

Considering a nonlinear time-discrete ***y*** *= g(****x****)* with mean $\bar{x}$ and covariance ***P***_***x***_, to calculate the statistics of ***y***, *2L+1* sigma points *χ*_*i*_ with its corresponding weighting factors is formulated as following equations

$$\{\begin{array}{cc} \chi_{0}=\bar{x} & i=0\\ \chi_{i}=\bar{x}+{\sqrt{\left(L+\lambda \right)P_{x}}}_{i} & \;i=1,\ldots \ldots ,L\\ \chi_{i}=\bar{x}-{\sqrt{\left(L+\lambda \right)P_{x}}}_{i-L} & i=L+1,\ldots \ldots ,2L\\ W_{0}^{\left(m\right)}=\lambda /\left(L+\lambda \right) & \\ W_{0}^{\left(c\right)}=\lambda /\left(L+\lambda \right)+1-\alpha^{2}+\beta & \\ W_{i}^{\left(m\right)}=W_{i}^{\left(c\right)}=0.5/\left(L+\lambda \right) & i=1,\;2,\ldots ,\;2L \end{array}$$ (23)

where *L* is the dimension of ***x***; *λ* = *α*^2^(*L* + *κ*) − *L* is a scaling parameter.*α* determines the spread of the sigma points around $\bar{x}$ and is usually set to a small positive value (*e*.*g*. 1e-3). *κ* is a secondary scaling parameter which is normally set to a positive value to ensure that the covariance matrix is positive definite. *β* is used to incorporate prior knowledge of the distribution of ***x***, which affects the weighting of the zero^th^ sigma point for the calculation of the covariance. For Gaussian distribution, *β* = 2 is optimal [22]. These sigma vectors are propagated through nonlinear function *y*_*i*_ = *g*(*χ*_*i*_), *i = 0*,*1*,*…2L*. The mean and covariance of ***y*** are estimated using the weighted sample mean and covariance of the posterior sigma points as follows,

$$\bar{y}=\sum_{i=0}^{2L}W_{i}^{\left(m\right)}y_{i}$$ (24)

$$P_{y}=\sum_{i=0}^{2L}W_{i}^{\left(c\right)}\left(y_{i}-\bar{y}\right){\left(y_{i}-\bar{y}\right)}^{T}$$ (25)

On the basis of unscented transform, the main steps of UKF are put forward:

1. Initialize vehicle state and covariance matrix at time step *k = 0* with
  $${\hat{x}}_{0}=E\left[x_{0}\right]$$ (26)
  $$P_{x0}=E[\left(x_{0}-{\hat{x}}_{0}\right)\cdot {\left(x_{0}-{\hat{x}}_{0}\right)}^{T}$$ (27)
2. For time step *k = 1*, *2 …*, calculate sigma points in sigma vector
  $$\chi \left(k-1\right)={\left[\begin{array}{c} \hat{x}\left(k-1\right)\\ \;\hat{x}\left(k-1\right)\;+\sqrt{\left(L+\lambda \right)P\left(k-1\right)}\\ \hat{x}\left(k-1\right)-\sqrt{\left(L+\lambda \right)P\left(k-1\right)} \end{array}\right]}^{T}$$ (28)
3. Time update
  - Propagate the sigma points through Eq (22).
    χ(k|k−1)=f(χ(k−1),u(k−1),w(k−1)) (29)
  - The propagated mean calculation
    $$\hat{x}\left(k|k-1\right)=\sum_{i=0}^{2L}W_{i}^{\left(m\right)}\cdot \chi_{i}\left(k|k-1\right)$$ (30)
  - The propagated covariance calculation
    $$P_{x}\left(k|k-1\right)=\sum_{i=0}^{2L}W_{i}^{\left(c\right)}\cdot [\chi_{i}\left(k|k-1\right)-\hat{x}\left(k|k-1\right)]\cdot {\left[\chi_{i}(k\left|k-1)-\hat{x}(k\right|k-1)\right]}^{T}+Q_{k}$$ (31)
4. Measurement update
  - Propagate sigma points through measurement function
    y(k|k−1)=h(χ(k−1),u(k−1),v(k−1)) (32)
  - The propagated mean calculation
    $$\hat{y}\left(k|k-1\right)=\sum_{i=0}^{2n}W_{i}^{\left(m\right)}\cdot y_{i}\left(k|k-1\right)$$ (33)
  - The propagated covariance and the Kalman gain calculation
    $$P_{y}\left(k|k-1\right)=\sum_{i=0}^{2n}W_{i}^{\left(c\right)}\cdot [y_{i}\left(k|k-1\right)-\hat{y}\left(k|k-1\right)]\cdot {\left[y_{i}(k\left|k-1)-\hat{y}(k\right|k-1)\right]}^{T}+R_{k}$$ (34)
    $$P_{xy}\left(k|k-1\right)=\sum_{i=0}^{2n}W_{i}^{\left(c\right)}\cdot [\chi_{i}\left(k|k-1\right)-\hat{x}\left(k|k-1\right)]\cdot {\left[y_{i}(k\left|k-1)-\hat{y}(k\right|k-1)\right]}^{T}$$ (35)
    $$K\left(k\right)=P_{xy}\left(k|k-1\right)\cdot P_{yy}{\left(k|k-1\right)}^{-1}$$ (36)
    where ***K****(k)* is the Kalman gain matrix.
  - Update the vehicle state estimation and state covariance
    $$\hat{x}\left(k|k\right)=\hat{x}\left(k|k-1\right)+K\left(k\right)\cdot \left[y\left(k\right)-\hat{y}\left(k|k-1\right)\right]$$ (37)
    $$P_{xx}\left(k|k\right)=P_{x}\left(k|k-1\right)-K\left(k\right)\cdot P_{y}\cdot K{\left(k\right)}^{T}$$ (38)

### Hybrid estimator design for tire-road friction coefficient

**Fig 3:**
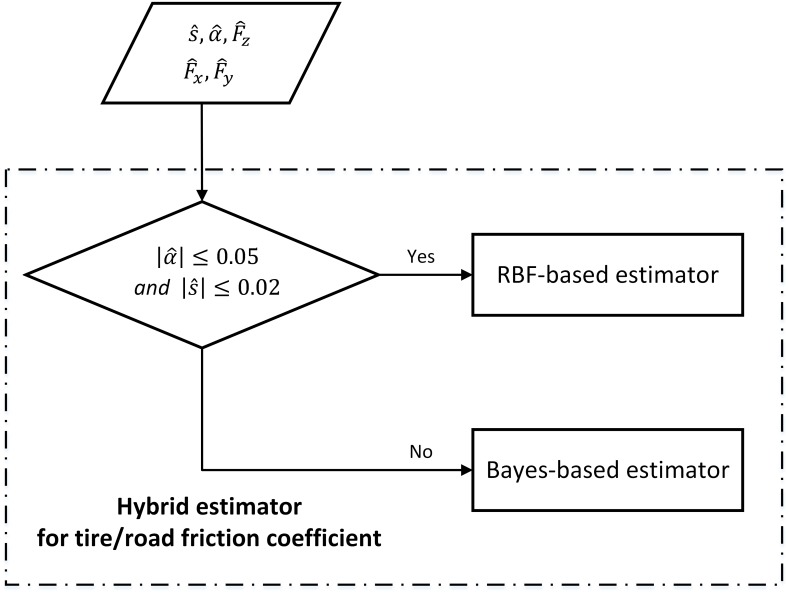
Structure of the hybrid estimator for TRFC.

The main objective of this section is to develop a robust TRFC estimator with a wide using range. It is obvious that appropriate excitations are very important for a TRFC estimation algorithm. However, since the excitation itself is just a response to road condition and driver behavior, the type and degree of excitations in vehicle applications are random at some point. Concerning this issue, a novel hybrid estimator consisting of two estimation algorithms is proposed according to the excitation levels, as shown in Fig 3. The TRFC estimation here is achieved through synthesizing the vehicle response to both longitudinal and lateral excitations instead of just one of them.

The main objective of this section is to develop a robust TRFC estimator with a wide using range. It is obvious that appropriate excitations are very important for a TRFC estimation algorithm. However, since the excitation itself is just a response to road condition and driver behavior, the type and degree of excitations in vehicle applications are random at some point. Concerning this issue, a novel hybrid estimator consisting of two estimation algorithms is proposed according to the excitation levels. As shown in Fig 3, $\hat{s}$, $\hat{\alpha }$, $\hat{F_{x}}$, $\hat{F_{y}}$ and $\hat{F_{z}}$ are the estimated slip ratio, slip angle, longitudinal, lateral and vertical force, respectively, from UKF estimator. Dynamic excitations acted on the vehicle are classified into small and large levels based on the estimated slip ratio and slip angle. The TRFC estimation here is achieved through synthesizing the vehicle response to both longitudinal and lateral excitations instead of just one of them.

#### GRNN-based estimator design

A GRNN is a powerful regression tool with a relatively simple network [23]. In this section it is applied to detect TRFC under small excitations, which are the most common situations in daily driving. Two main benefits from this method are that firstly a GRNN can establish network connections between input and output instead of storing an entire complex tire model in the controller, which can significantly reduce the computations and guarantee the real time performance; secondly because the GRNN is trained by measured data, it is able to accurately create a mapping from input parameters to the friction coefficient [24]. Besides, it should be noted that a successful training of a neural network needs a data set that traverses all driving conditions, which is difficult to be achieved. Nevertheless since the proposed GRNN estimator only serves for small excitations conditions, the range of input parameters is limited, which objectively makes it possible to acquire the data that just comprises the relevant conditions to train the network.

As previously stated, in this article the step of data collecting is conducted according to “Pacejka 2002” tire model. In the data generation process, friction coefficient, normal tire load, slip ratio, and slip angle are taken as the input of the tire model and then longitudinal and lateral tire forces can be calculated. Under the precondition of small excitations, the range of variation of the input parameters to the tire model is bounded as shown in Table 1. Additionally, the distribution of the given inputs is independent with each other.

**Table 1:** Ranges of Input Parameters.

Input parameter	Range of variation
Tire-road friction coefficient	0.1 to 1 at intervals of 0.1
Normal load *F*_*z*_	[1700 4500] *N*
Slip ratio *λ*	[-2 2] *%*
Slip angle *α*	[-0.05 0.05] *rad*

**Fig 4:**
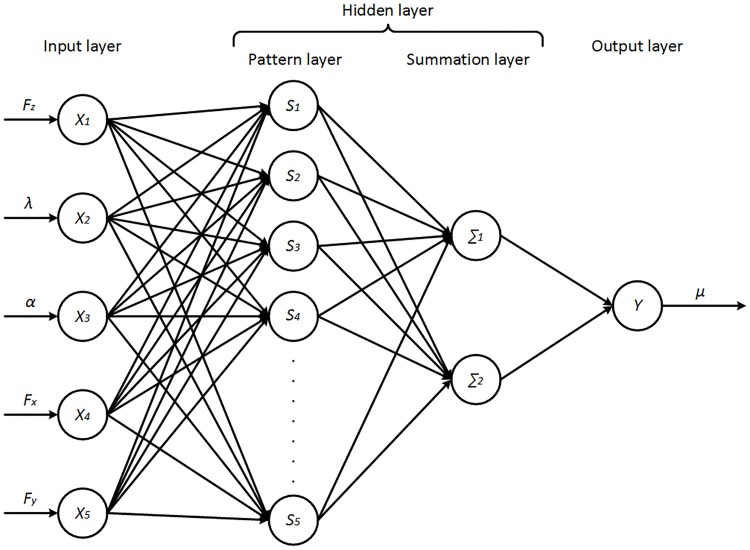
GRNN architecture used for the TRFC estimation.

About 100,000 original data are obtained from data collecting stage. Two-thirds of the collected data are randomly taken as the training set and the rest as the test set. The data of *F*_*z*_, *λ*, *α*, *F*_*x*_, and *F*_*y*_ are fed into the neural network, while the TRFC is set as the output of the neural network. Fig 4 shows the GRNN architecture used for the TRFC estimation.

**Fig 5:**
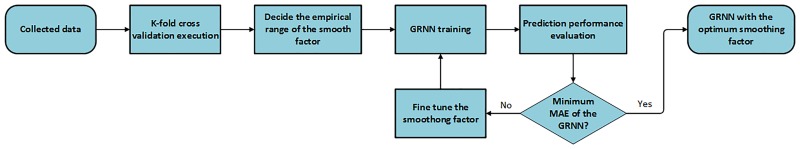
The process of GRNN establishment.

**Fig 6:**
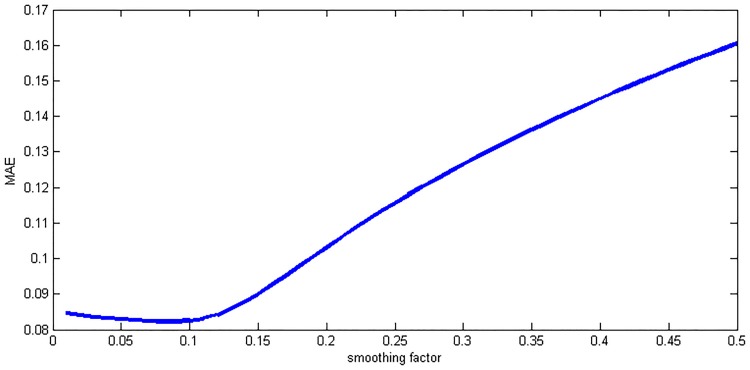
The selection of the smoothing factor.

The GRNN regression formula is given as:

$$\hat{Y}\left(X\right)=\frac{\sum_{i=1}^{n}Y^{i}\text{exp}\left(-\frac{{\left(X-X^{i}\right)}^{T}\left(X-X^{i}\right)}{2\sigma^{2}}\right)}{\sum_{i=1}^{n}\text{exp}\left(-\frac{{\left(X-X^{i}\right)}^{T}\left(X-X^{i}\right)}{2\sigma^{2}}\right)}$$ (39)

where *X* is the independent input variables and *Y* is corresponding output. Moreover, in the network the smoothing factor *σ* is the only parameter that can be adjusted. It determines the generalization ability of the network. When *σ* is made large, the estimated density is forced to be smooth and in the limit becomes a multivariate Gaussian with covariance *σ*^2^ · ***I*** (unit matrix), while a smaller *σ* allows the estimated density to assume non-Gaussian shapes, but with the hazard that wild points may have a great effect on the estimate [23]. It is therefore necessary for GRNN modeling to find the optimum smoothing factor. The whole process of GRNN establishment is illustrated in Fig 5, where K-fold cross validation [25] is applied to calculate an appropriate smoothing parameter at which the mean absolute error (MAE) of the network was the lowest [26]. As shown in Fig 6 the optimal smoothing factor is finally set as 0.082.

#### Bayesian theorem-based estimator design

The GRNN-based algorithm cannot perform well if the tire slip ratio or slip angle is beyond the limited range. Bayes-based estimator is designed to extend the range of RBF-based estimator. Estimated forces from UKF are firstly normalized by their respective normal forces and then compared with those from a nominal tire model to determine the most probable friction coefficient from a set of hypothesized values [12, 27].

The estimated forces of each wheel from UKF are normalized as follows,

$${\hat{\varphi }}_{i}=\frac{{\left[\begin{array}{cc} {\hat{F}}_{ti} & {\hat{F}}_{si} \end{array}\right]}^{T}}{{\hat{F}}_{zi}}$$ (40)

*i = fl*, *fr*, *rl* and *rr* represents the front left, front right, rear left and rear right wheel.

In addition, the tire forces according to the nominal “Pacejka 2002” tire model is denoted by ${\hat{\psi }}_{i,j}$,

$${\hat{\psi }}_{i,j}=\frac{PAC{\left(\hat{s},\;\hat{\alpha },{\hat{F}}_{zi},\mu_{i,j}\right)}^{T}}{{\hat{F}}_{zi}},\;j=1,2,3\ldots \ldots 10$$ (41)

where *j* represents the set of hypothesized friction coefficients.

Then likelihood function of *μ*_*i*,*j*_ is

$$\begin{array}{l} \;L\left(\mu_{i,j}|{\hat{\varphi }}_{i}\right)=p\left({\hat{\varphi }}_{i}|\mu_{i,j}\right)\\ =p\left({\hat{\varphi }}_{i}|{\hat{\psi }}_{i,j}\right)=\frac{1}{2\pi \cdot {\left|\Sigma \right|}^{1/2}}exp\left[-\frac{1}{2}{\left({\hat{\varphi }}_{i}-{\hat{\psi }}_{i,j}\right)}^{T}V^{-1}\left({\hat{\varphi }}_{i}-{\hat{\psi }}_{i,j}\right)\right] \end{array}$$ (42)

Eq (42) describes the estimation of parameter *μ*_*i*,*j*_ for a given outcome ${\hat{\varphi }}_{i}$. In this equation **Ʃ** is a 2 × 2 covariance matrix. $p\left({\hat{\varphi }}_{i}|\mu_{i,j}\right)$ is the probability density of obtaining ${\hat{\varphi }}_{i}$ under a given TRFC *μ*_*i*,*j*_.

The prior probability of road-tire coefficient *μ*_*i*,*j*_ is defined *P*_0_(*μ*_*i*,*j*_) and equals to $\frac{1}{10}$.

On basis of Bayes' theorem, at sampling time *t*_*k*_, the conditional probability of *μ*_*i*,*j*_ under the estimated ${\hat{\varphi }}_{k,i}$ is given as

$$P_{k+1}\left(\mu_{i,j}|{\hat{\varphi }}_{k+1,i}\right)=\frac{p\left({\hat{\varphi }}_{k,i}|\mu_{i,j}\right)\cdot P_{k}(\mu_{i,j})}{\sum_{j=1}^{10}p\left({\hat{\varphi }}_{k,i}|\mu_{i,j}\right)\cdot P_{k}(\mu_{i,j})},\;k=0,1,2\ldots \ldots$$ (43)

The current TRFC is calculated by a weighted sum

$${\hat{\mu }}_{k}=\sum_{j=1}^{10}P_{k+1}\left(\mu_{i,j}|{\hat{\varphi }}_{k+1,i}\right)\cdot \mu_{i,j}$$ (44)

At the next sampling time *t*_*k*+1_, by repeating the above process, the online friction coefficient estimation for large excitations can be achieved.

## Simulation results

The simulation presented in this section is carried out through the co-simulation of Matlab/Simulink and CarSim. It should be noted that a detailed vehicle model in CarSim involves a full-vehicle multibody dynamics model (including a closed-loop driver model, powertrain system, brake system and “Pacejka 2002” tire model, etc.) that is much more complex and complete than the model used for UKF estimator design. Therefore, the CarSim vehicle model is used to simulate a real vehicle, provide reference vehicle state and measured signals, while the estimation algorithms are built in Matlab/Simulink environment. Moreover, Gaussian noises are added in the simulated measurements to realistically represent real application. The vehicle parameters used in the simulation are listed in Table 2. 5% differences of these parameters are added to the UKF 3-DOF vehicle model in the simulation to imitate modelling uncertainties.

**Table 2:** Vehicle parameters.

Parameter	Unit	Value
Gross Mass	*m (kg)*	1280
Height of sprung mass center of gravity	*hg (m)*	0.5
Distance from COG to front wheels	*a (m)*	1.203
Distance from COG to rear wheels	*b (m)*	1.217
Wheelbase	*l (m)*	2.420
Wheel track	*T (m)*	1.330
Wheel Radius	*R (m)*	0.298
Vehicle rotational inertia about Z-axis	*I*_*z*_ *(kg·m*^*2*^*)*	2500
Tire rotational inertia	*I*_*r*_ *(kg·m*^*2*^*)*	2.5

Two maneuvers are conducted to evaluate the proposed TRFC estimation algorithm performance under various vehicle movements such as acceleration, deceleration, constant speed, and steering.

### Acceleration and brake maneuver

**Fig 7:**
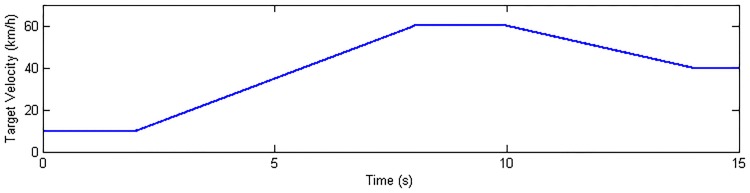
Target velocity in CarSim.

**Fig 8:**
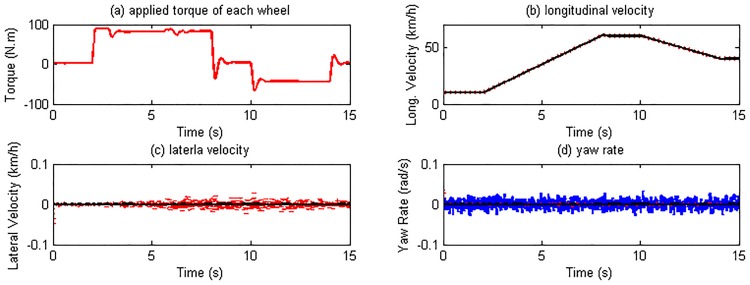
Control input and vehicle state estimation. (The red dotted line is estimated value; continuous black line is reference value; continuous blue line is sensor noise.)

The longitudinal performance of the proposed estimation algorithm will be investigated and analyzed under acceleration and brake maneuver. The road surface input in CarSim is set according to Table 3. The driver model embedded in CarSim controls the vehicle to follow the given target velocity as shown in Fig 7. For simplification, the driver desired driving and braking torque are distributed equally on four wheels as the control input shown in Fig 8(a). Fig 8(b) and 8(c) show the estimation results of vehicle response. It can be seen that the longitudinal velocity is estimated extremely well. The errors of lateral velocity and yaw rate are also acceptable.

**Table 3:** Road surface in CarSim.

Station (m)	Friction coefficient
0–30	0.9
30–40	0.3
40–80	0.5
80–140	0.7
140-end	0.4

**Fig 9:**
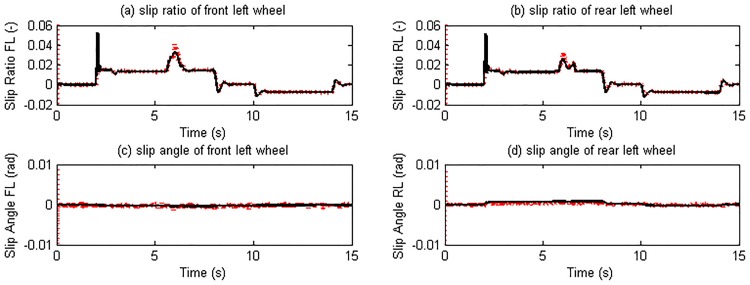
Slip ratio and slip angle estimation results. (The red dotted line is estimated value; continuous black line is reference value).

**Fig 10:**
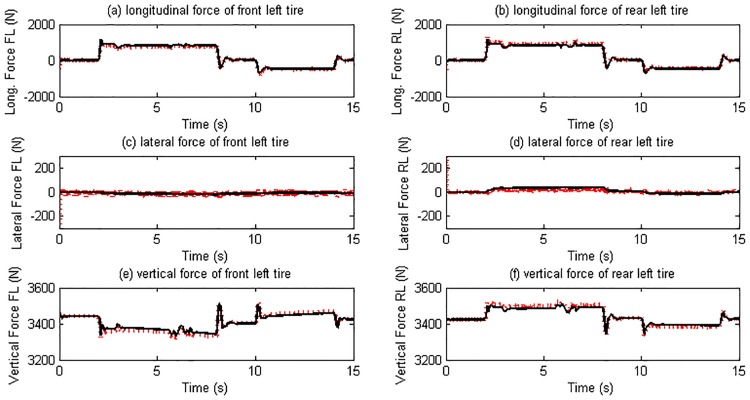
Tire force estimation results. (The red dotted line is estimated value; continuous black line is reference value).

As this maneuver forces on vehicle longitudinal motion, the response of the right and left side tires is similar to each other. Therefore here we only list the simulation results of the left side tires. The estimated slip ratios, slip angles, and tire forces are illustrated in Figs 9 and 10.

**Fig 11:**
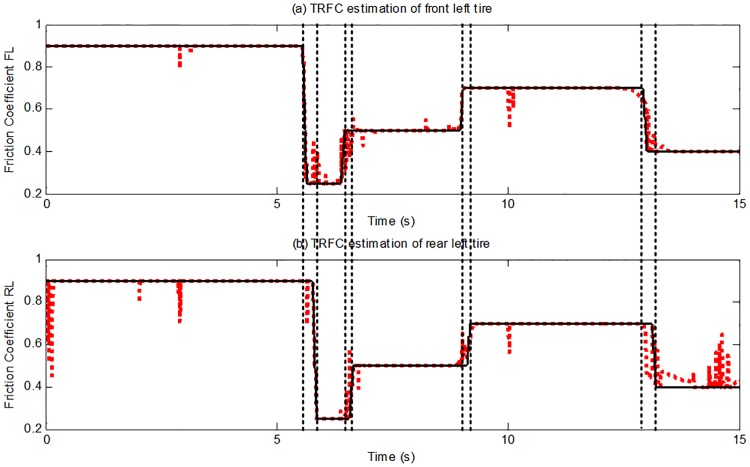
Tire-road friction coefficient estimation. (The red dotted line is estimated value; continuous black line is reference value).

Fig 11 presents the estimation results and reference values of the TRFC. When the road surface changes, the estimation converges to the reference value accurately and rapidly, as can be seen in Fig 11. Additionally, the changes of the road frictional conditions for the front and rear wheels are sequentially identified. The simulation results clearly demonstrate that the proposed estimation method is reliable and applicable on a straight ahead driving maneuver.

### Double line change maneuver

**Fig 12:**
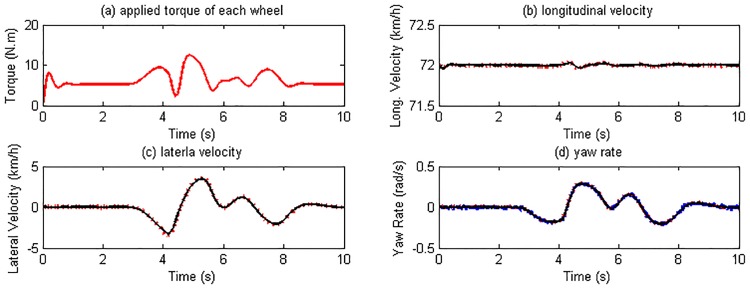
Control input and vehicle state estimation. (The red dotted line is estimated value; continuous black line is reference value; continuous blue line is sensor noise.)

**Fig 13:**
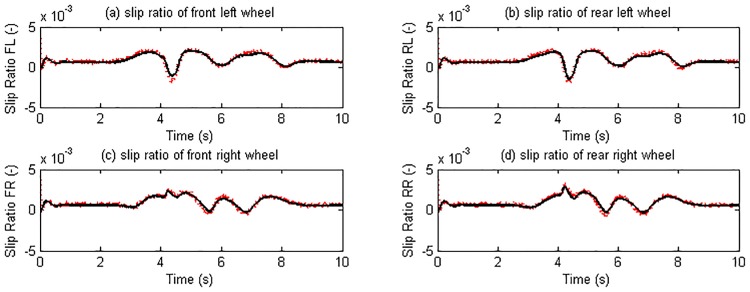
Slip ratio estimation results. (The red dotted line is estimated value; continuous black line is reference value).

**Fig 14:**
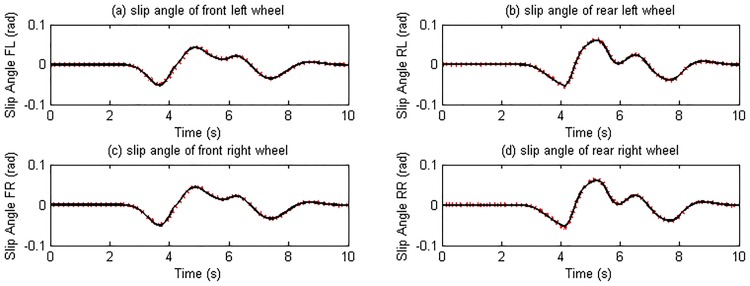
Slip angle estimation results. (The red dotted line is estimated value; continuous black line is reference value).

**Fig 15:**
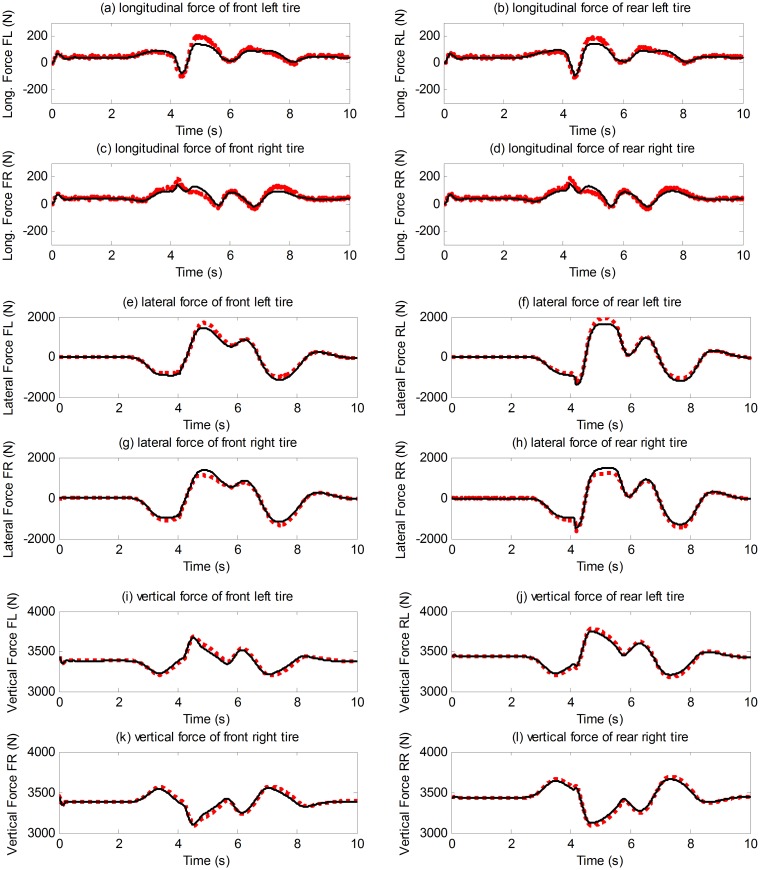
Tire force estimation results. (The red dotted line is estimated value; continuous black line is reference value).

The double change maneuver is conducted to verify the proposed estimation method on steering condition. In the simulation, the vehicle speed is maintained at 72 km/h and the TRFC in CarSim is set according to Table 4. The driver desired torque from CarSim driver model is distributed equally on four wheels as shown in Fig 12(a). The estimation results of longitudinal velocity, lateral velocity, yaw rate, slip ratio, slip angle, tire force are presented in Figs 13–15. It is indicated that the estimation algorithm is reliable and accurate; the UKF estimator and the TRFC estimator are able to mutually effect and correct each other.

**Table 4:** Road surface in CarSim.

Station (m)	Friction coefficient
0–40	0.8
40–80	0.3
80-end	0.5

**Fig 16:**
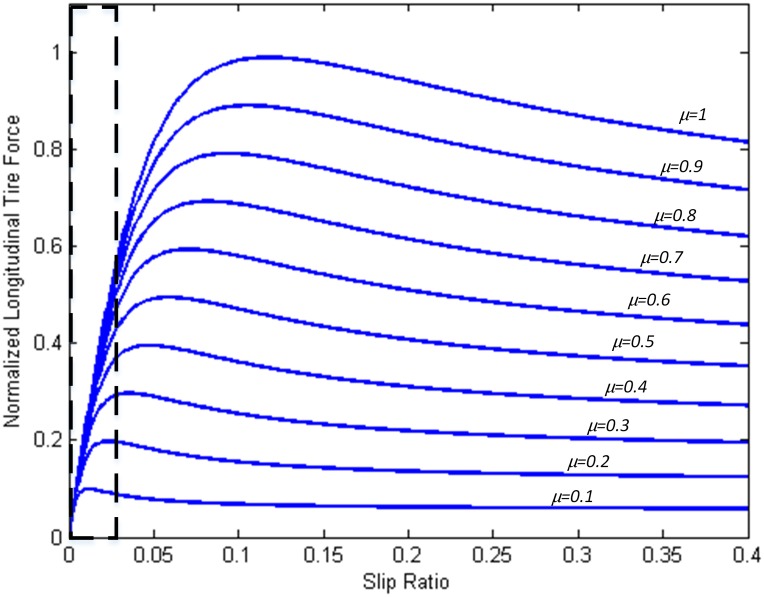
Relationship between slip ratio and normalized longitudinal tire force under different friction coefficients.

It is noteworthy that a relatively large estimation error appears at the beginning of this maneuver, which is believed to be caused by the inadequate excitation due to the uniform straight line motion of the vehicle. As we know, when the vehicle is in straight line motion, the excitation level is reflected only through slip ratio. Fig 16 is given showing the relationship between the slip ratio and the normalized longitudinal tire force under different friction coefficient *μ*. It can be seen that in the dash line marked area the spacing between all the curves is quite close. This spacing becomes even smaller as the slip ratio decreases. If the slip ratio is approximate to zero, such as under the uniform straight line motion at the beginning of this double line change maneuver, we will see basically no distinction between the vehicle longitudinal dynamic responses under different road conditions. Meanwhile, this vehicle does not have any lateral dynamic response. Therefore, it is extremely difficult for the TRFC estimator to distinguish the road surface conditions.

**Fig 17:**
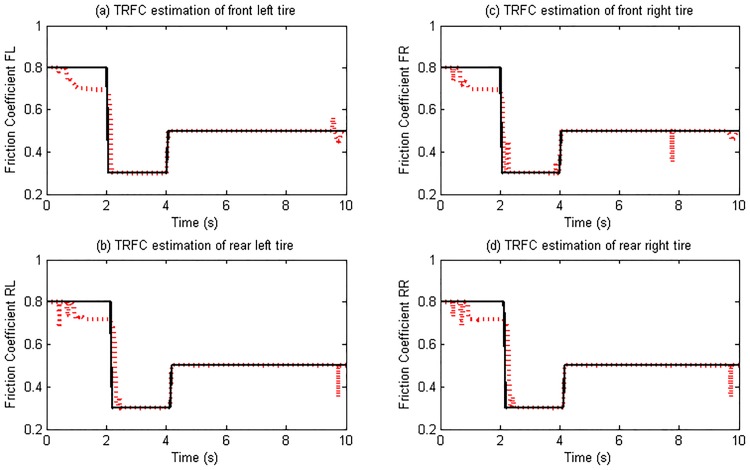
Tire-road friction coefficient estimation. (The red dotted line is estimated value; continuous black line is reference value).

Despite this challenging testing situation, the estimation results are also acceptable. Once the steering operation is implemented, the estimated friction coefficient quickly converges to the reference values, as can be seen in Fig 17. This quick convergence is due to the lateral dynamic response of the vehicle led by steering maneuver. Then the TRFC estimation can be carried out synthesizing both longitudinal and lateral excitations instead of just one of them, which validates the effectiveness of the designed estimation method.

## Conclusion

In this study, we presented a hierarchical TRFC estimation method based on a combination of UKF estimator, GRNN and Bayes theorem, in which UKF estimator severs for vehicle dynamic states estimation; GRNN and Bayes theorem are applied for TRFC estimation under small and large excitation as a hybrid estimator, respectively. The overall estimation algorithm was evaluated on varying road surfaces through the co-simulation environment of Matlab/Simulink and CarSim. The simulation results show that the estimation has favorable coincidence with the corresponding reference values.

Further research may focus in the following aspects:

1. The estimation of vehicle parameters (such as mass, rotational inertia, etc.) should be taken into account in further research.
2. Since the proposed method is only analyzed theoretically and validated via simulation, an actual bench or field test is needed in the future to verify the proposed approach.

## Supporting information

S1 Table The simulation data of acceleration and brake maneuver. (XLSX)

S2 Table The simulation data of double line change maneuver. (XLSX)

## References

[1] Van Zanten AT. Bosch ESP Systems: 5 Years of Experience. SAE International; 2000.

[2] Cho K, Kim J, Choi S, editors. The integrated vehicle longitudinal control system for ABS and TCS. Control Applications (CCA), 2012 IEEE International Conference on; 2012: IEEE.

[3] He H, Peng J, Xiong R, Fan H. An Acceleration Slip Regulation Strategy for Four-Wheel Drive Electric Vehicles Based on Sliding Mode Control. Energies. 2014;7(6):3748–63.

[4] Müller S, Uchanski M, Hedrick K. Estimation of the Maximum Tire-Road Friction Coefficient. Journal of Dynamic Systems, Measurement, and Control. 2004;125(4):607–17.

[5] Holzmann F, Bellino M, Siegwart R, Bubb H, editors. Predictive estimation of the road-tire friction coefficient. Computer Aided Control System Design, 2006 IEEE International Conference on Control Applications, 2006 IEEE International Symposium on Intelligent Control, 2006 IEEE; 2006: IEEE.

[6] Riehm M, Gustavsson T, Bogren J, Jansson P-E. Ice formation detection on road surfaces using infrared thermometry. Cold Regions Science and Technology. 2012;83:71–6.

[7] Ahn C, Peng H, Tseng HE, editors. Estimation of road friction for enhanced active safety systems: Algebraic approach. American Control Conference, 2009 ACC'09; 2009: IEEE.

[8] Rajamani R, Piyabongkarn N, Lew J, Yi K, Phanomchoeng G. Tire-road friction-coefficient estimation. IEEE Control Systems. 2010;30(4):54–69.

[9] Wenzel TA, Burnham K, Blundell M, Williams R. Dual extended Kalman filter for vehicle state and parameter estimation. Vehicle System Dynamics. 2006;44(2):153–71.

[10] Antonov S, Fehn A, Kugi A. Unscented Kalman filter for vehicle state estimation. Vehicle System Dynamics. 2011;49(9):1497–520.

[11] Ren H, Chen S, Shim T, Wu Z. Effective assessment of tyre–road friction coefficient using a hybrid estimator. Vehicle System Dynamics. 2014;52(8):1047–65.

[12] Ray LR. Nonlinear tire force estimation and road friction identification: Simulation and experiments. Automatica. 1997;33(10):1819–33.

[13] Zareian A, Azadi S, Kazemi R. Estimation of road friction coefficient using extended Kalman filter, recursive least square, and neural network. Proceedings of the Institution of Mechanical Engineers, Part K: Journal of Multi-body Dynamics. 2016;230(1):52–68.

[14] Hsu Y-HJ, Gerdes JC, editors. A feel for the road: A method to estimate tire parameters using steering torque. International Symposium on Advanced Vehicle Control; 2006.

[15] Pingping GHWBL, Liang X. Identification of maximum road friction coefficient and optimal slip ratio based on road type recognition. Chinese Journal of Mechanical Engineering. 2014;27(05):1.

[16] Chen Y, Wang J. Adaptive vehicle speed control with input injections for longitudinal motion independent road frictional condition estimation. IEEE Transactions on Vehicular Technology. 2011;60(3):839–48.

[17] Wang R, Wang J. Tire–road friction coefficient and tire cornering stiffness estimation based on longitudinal tire force difference generation. Control Engineering Practice. 2013;21(1):65–75.

[18] Esmailzadeh E, Vossoughi G, Goodarzi A. Dynamic modeling and analysis of a four motorized wheels electric vehicle. Vehicle System Dynamics. 2001;35(3):163–94.

[19] Pacejka H. Tire and vehicle dynamics: Elsevier; 2005.

[20] Kandepu R, Foss B, Imsland L. Applying the unscented Kalman filter for nonlinear state estimation. Journal of process control. 2008;18(7):753–68.

[21] Julier SJ, Uhlmann JK, editors. New extension of the Kalman filter to nonlinear systems. AeroSense'97; 1997: International Society for Optics and Photonics.

[22] Wan EA, Van Der Merwe R, editors. The unscented Kalman filter for nonlinear estimation. Adaptive Systems for Signal Processing, Communications, and Control Symposium 2000 AS-SPCC The IEEE 2000; 2000: Ieee.

[23] Specht DF. A general regression neural network. IEEE transactions on neural networks. 1991;2(6):568–76. doi: 10.1109/72.97934 18282872 10.1109/72.97934

[24] Pasterkamp WR, PACEJKA HB. The tyre as a sensor to estimate friction. Vehicle System Dynamics. 1997;27(5–6):409–22.

[25] Arlot S, Celisse A. A survey of cross-validation procedures for model selection. Statistics surveys. 2010;4:40–79.

[26] Gao S, Tian J, Wang F, Bai Y, Gao W, Yang S, editors. The Study of GRNN for Wind Speed Forecasting Based on Markov Chain. Proceedings of International Conference on Modelling, Simulation and Applied Mathematics (MSAM 2015); 2015.

[27] Ray LR, editor Real time determination of road coefficient of friction for IVHS and advanced vehicle control. American Control Conference, Proceedings of the 1995; 1995: IEEE.

